# Supplementary material for: ALKBH5 modulation of ferroptosis in recurrent miscarriage: implications in cytotrophoblast dysfunction
Source: PeerJ. 2024 Oct 18;12:e18227. doi: 10.7717/peerj.18227 (PMC11493020; doi:10.7717/peerj.18227)
Supplement: Supplemental Information 1 [file peerj-12-18227-s001.docx]

Supplemental Table 1. Primers used for qPCR in the present study

| Primer name | Primer sequence (5′ to 3′) |
| --- | --- |
| METTL3-qPCR-F | CTATCTCCTGGCACTCGCAAGA |
| METTL3-qPCR-R | GCTTGAACCGTGCAACCACATC |
| METTL14-qPCR-F | CTGAAAGTGCCGACAGCATTGG |
| METTL14-qPCR-R | CTCTCCTTCATCCAGATACTTACG |
| WTAP-qPCR-F | GCAACAACAGCAGGAGTCTGCA |
| WTAP-qPCR-R | CTGCTGGACTTGCTTGAGGTAC |
| ALKBH5-qPCR-F | CCAGCTATGCTTCAGATCGCCT |
| ALKBH5-qPCR-R | GGTTCTCTTCCTTGTCCATCTCC |
| FTO-qPCR-F | CCAGAACCTGAGGAGAGAATGG |
| FTO-qPCR-R | CGATGTCTGTGAGGTCAAACGG |
| YTHDF1-qPCR-F | CAAGCACACAACCTCCATCTTCG |
| YTHDF1-qPCR-R | GTAAGAAACTGGTTCGCCCTCAT |
| YTHDF2-qPCR-F | TAGCCAGCTACAAGCACACCAC |
| YTHDF2-qPCR-R | CAACCGTTGCTGCAGTCTGTGT |
| YTHDF3-qPCR-F | GCTACTTTCAAGCATACCACCTC |
| YTHDF3-qPCR-R | ACAGGACATCTTCATACGGTTATTG |
| FTL-qPCR-F | TACGAGCGTCTCCTGAAGATGC |
| FTL-qPCR-R | GGTTCAGCTTTTTCTCCAGGGC |
| β-actin-qPCR-F | CACCATTGGCAATGAGCGGTTC |
| β-actin-qPCR-R | AGGTCTTTGCGGATGTCCACGT |
