# Supplementary figures and images for: ALKBH5 modulation of ferroptosis in recurrent miscarriage: implications in cytotrophoblast dysfunction
Source: PeerJ. 2024 Oct 18;12:e18227. doi: 10.7717/peerj.18227 (PMC11493020; doi:10.7717/peerj.18227)

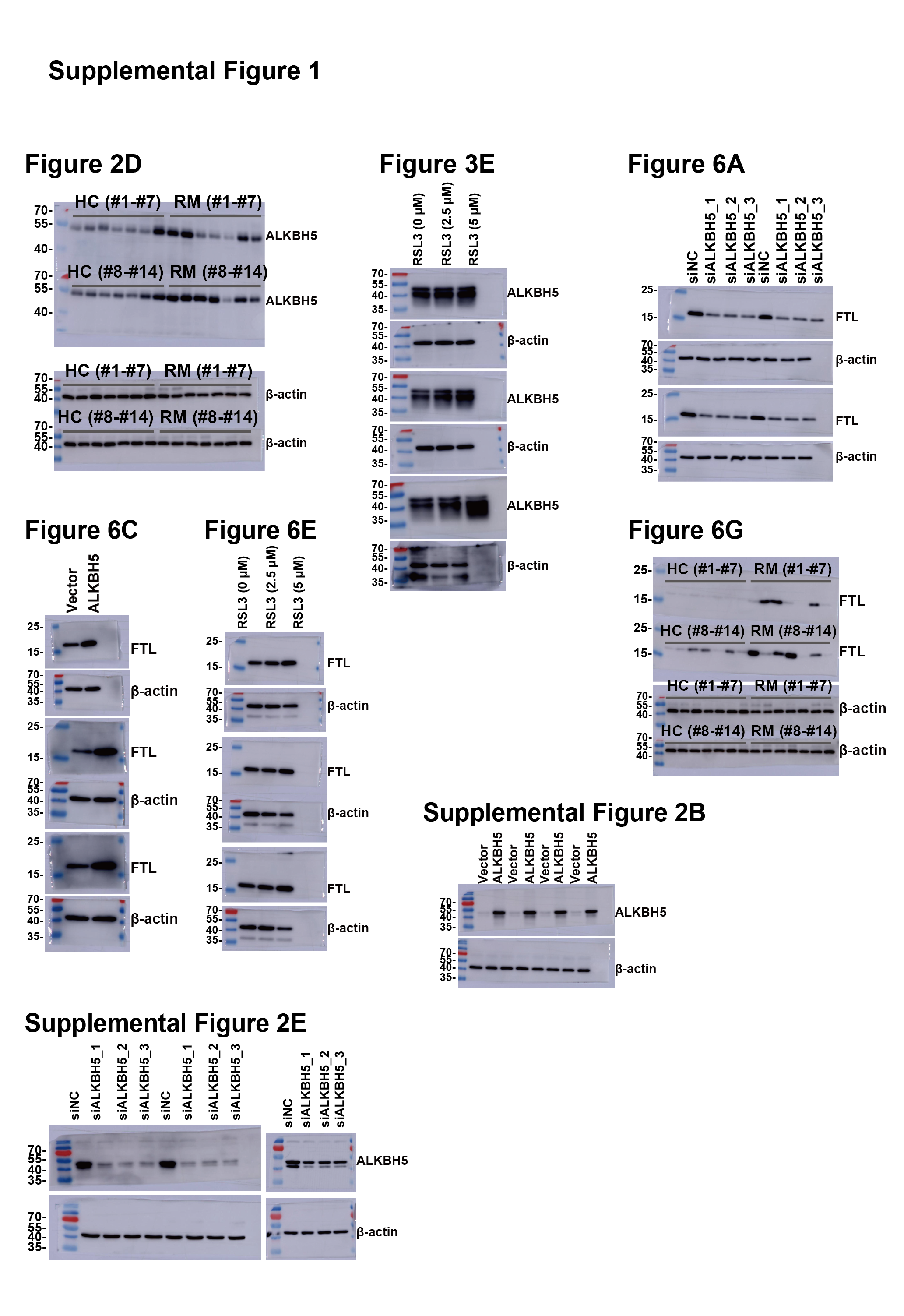

Supplement: Supplemental Information 2 [file peerj-12-18227-s002.png]

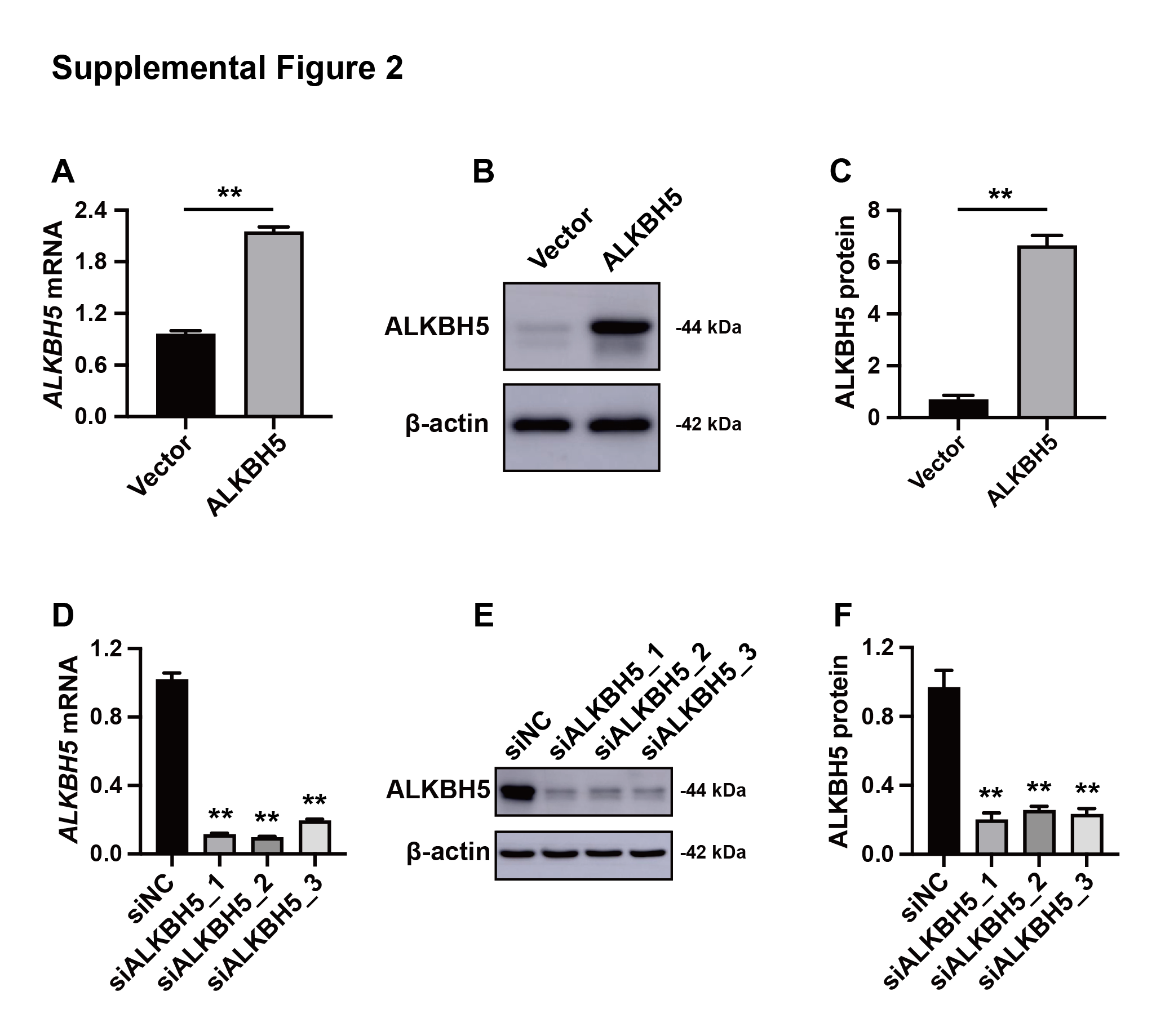

Supplement: Supplemental Information 3 — (A) qRT-PCR results showing ALKBH5 mRNA levels in HTR8 cells transfected with pLV or pLV-ALKBH5. (B, C) Western blotting results showing ALKBH5 levels in HTR8 cells transfected with pLV or pLV-ALKBH5. (D) qRT-PCR results showing ALKBH5 mRNA levels in HTR8 cells transfected with siNC or siALKBH5. (E, F) Western blotting results showing ALKBH5 levels in HTR8 cells transfected with siNC or siALKBH5. (**p < 0.01). [file peerj-12-18227-s003.png]
